# Virtual Screening of FDA-Approved Drugs against LasR of *Pseudomonas aeruginosa* for Antibiofilm Potential

**DOI:** 10.3390/molecules25163723

**Published:** 2020-08-15

**Authors:** Suhaib Sadiq, Nosheen Fatima Rana, Muhammad Ammar Zahid, Muhammad Kazim Zargaham, Tahreem Tanweer, Amna Batool, Ayesha Naeem, Afrah Nawaz, Rizwan-ur-Rehman, Zahid Muneer, Abdul Rauf Siddiqi

**Affiliations:** 1 Department of Biomedical Engineering and Sciences, School of Mechanical and Manufacturing Engineering, National University of Sciences & Technology, Islamabad 44000, Pakistan; suhaibsadiq.ss@gmail.com (S.S.); ttanveer.pg@smme.edu.pk (T.T.); amnabatoolx@gmail.com (A.B.); ayesha.n.mehmood@gmail.com (A.N.); afrahnawaz@yahoo.com (A.N.); 2 Shifa College of Pharmaceutical Sciences, Shifa Tameer-e-Millat University, Islamabad 44000, Pakistan; ammar.scps@stmu.edu.pk (M.A.Z.); kazim.scps@stmu.edu.pk (M.K.Z.); 3 School of Food and Agricultural Sciences, University of Managenent and Technology, Main Campus Lahore 54000, Pakistan; Rizwan.rehman@umt.edu.pk; 4 Department of Bioscience, COMSATS University Islamabad, Park Road Islamabad 44000, Pakistan; zahid.muneer@comsats.edu.pk

**Keywords:** LasR inhibitors, virtual screening, pharmaco-informatics

## Abstract

*Pseudomonas aeruginosa* is a Gram-negative pathogenic bacterium that is present commonly in soil and water and is responsible for causing septic shock, pneumonia, urinary tract and gastrointestinal infections, etc. The multi-drug resistance (MDR) phenomenon has increased dramatically in past years and is now considered a major threat globally, so there is an urgent need to develop new strategies to overcome drug resistance by *P. aeruginosa*. In *P. aeruginosa*, a major factor of drug resistance is associated to the formation of biofilms by the LasR enzyme, which regulates quorum sensing and has been reported as a new therapeutic target for designing novel antibacterial molecules. In this study, virtual screening and molecular docking were performed against the ligand binding domain (LBD) of LasR by employing a pharmacophore hypothesis for the screening of 2373 FDA-approved compounds to filter top-scoring hit compounds. Six inhibitors out of 2373 compounds were found to have binding affinities close to that of known LasR inhibitors. The binding modes of these compounds to the binding site in LasR-LBD were analyzed to identify the key interactions that contribute to the inhibition of LasR activity. Then, 50 ns simulations of top hit compounds were performed to elucidate the stability of their binding conformations with the LasR-LBD. This study, thus concluded that sulfamerazine showed the highest binding affinity for the LasR-LBD binding pocket exhibiting strong inhibitory binding interactions during molecular dynamics (MD) simulation.

## 1. Introduction

*Pseudomonas aeruginosa* is a well-known opportunistic bacterium that is responsible for many nosocomial infections worldwide, accounting for approximately 10% of all infections in European Union hospitals [1]. It causes respiratory tract infections, urinary tract infections, dermatitis, chronic wounds, soft tissue infections, and several systemic infections specifically in patients with comorbid severe burns, cancer, and AIDS patients who are already immunocompromised. Resistance towards antimicrobial agents is a widely studied research problem, and *Pseudomonas aeruginosa* is one of the bacteria that shows resistance towards antimicrobial agents by forming biofilms and results in chronic infections [2]. In fact, biofilm-forming bacteria are 100–1000 times more resistant to antimicrobial agents [3].

Biofilms formed by *Pseudomonas aeruginosa* are heterogeneous and mushroom-shaped microcolonies and use carbon as a source of nutrients. The persistence of chronic *Pseudomonas aeruginosa* lung infections in cystic fibrosis (CF) patients is due to alginate producing mucoid strains grown by biofilm. The biofilm serves as armor for the bacteria, embedded in a self-synthesized polymer matrix consisting of polysaccharides, proteins, and DNA [4]. Due to its complex nature, researchers have tried several strategies to block biofilm forming molecular cascades, but, remarkably, a solution is still wanting. Herein we focus on the molecules that target quorum sensing (QS) which has been proposed as an anti virulence strategy.

In *Pseudomonas aeruginosa*, QS is considered as a key mechanism in the regulation of virulence factor production and in the formation of biofilms that make it tolerant to antimicrobials. The signal N-3-oxododecanoyl homoserine lactone (3OC12-HSL) is produced by a synthase called LasI (encoded by lasI). The 3OC12-HSL signal affects gene expression by binding to a specific signal receptor called LasR (encoded by *lasR*), which in turn modulates transcription of effector genes. LasI and LasR are members of conserved families of synthases and receptors that have been found in dozens of different species of Proteobacteria. Acyl-homoserine lactone (acyl-HSL) signals produced by homologs of LasI differ in their acyl side chains, and the receptors have differing specificities for the various acyl-HSLs [5,6,7,8]. The LasRI system controls the expression of many genes, several of which encode virulence factors. Mutants with defects in quorum sensing (rhlI, lasI, and lasR) have substantially reduced virulence in a variety of animal models. Furthermore, there is evidence that inhibitors of 3OC12-HSL quorum sensing can reduce the severity and duration of *P. aeruginosa* lung infections in rodents. The second acyl-HSL signaling system in *P. aeruginosa*, the RhlR-RhlI system (encoded by rhlR and rhlI), also controls the expression of multiple genes. Production of RhlR and RhlI requires activation by the LasR-LasI system. Thus, the LasR-LasI system is at the top of the acyl-HSL signaling cascade in *P. aeruginosa*. There is also a third LasR homolog called QscR that lacks a cognate I protein. Instead, like LasR, it responds to 3OC12-HSL and regulates a set of genes that overlap with the LasR- and RhlR-activated genes [9,10,11,12].

**Figure 1:**
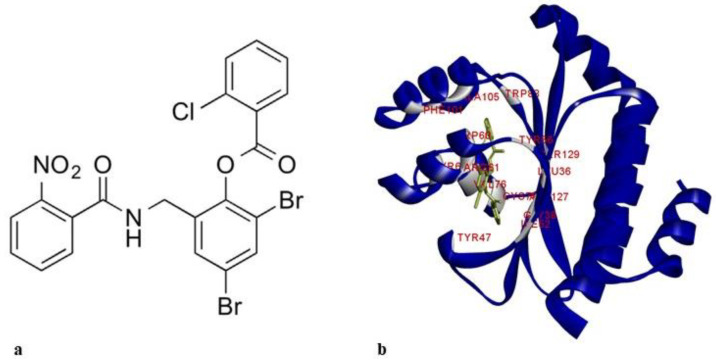
(**a**) 2D structure of TP1; (**b**) LasR-LBD:TP1 complex (3D structure showing the interaction of TP1 and LBD). Amino acids involved in interaction are labeled and colored in silver. LBD: ligand binding domain.

LasR comprises two independently folded protein domains: an amino-terminal ligand-binding domain (LBD) and a carboxy-terminal DNA-binding domain. Binding of homoserine lactone autoinducer promotes the dimerization of two LasR subunits. The resulting ligand-bound homodimer binds target DNA to activate gene transcription [13]. The structure of LasR-LBD (Protein Data Bank (PDB) code: 6D6A) with the known ligand, TP1, is shown in Figure 1.

Native autoinducers and already-reported inhibitors bind to the LBD of LasR, so this study was focused on the LBD. Virtual screening techniques are cost effective and faster, whereby large compound libraries can be screened to discover new hit molecules based on their structure that are most likely to bind to a drug target. With our objective of identifying new ligand molecules that will bind selectively to LBD, we adopted a combined structure and ligand-based virtual screening protocol entailing pharmacophore modeling, molecular docking, and molecular dynamics (MD) simulations.

## 2. Results

### 2.1. Description of Pharmacophore

Three common pharmacophore hypotheses (CPH) were generated using the PHASE module with the help of 31 reported LasR-LBD inhibitors. A characteristic framework was used to create a list of processes that helped in developing pharmacophore models since the modelling process was qualitative in nature [14]. For distinguishing active from inactive, a threshold IC_50_ of 30 µM was used. Three different hypotheses were identified (Table 1).

All the generated hypotheses were then submitted to a PHASE scoring procedure, which consisted of survival active and survival inactive scores. PhaseHypoScore ranks hypotheses by their likely performance in virtual screening as well as the quality of ligand alignment. It easily recognizes multiple binding modes in hypotheses from common pharmacophore perceptions when training against diverse known actives. ADHRR was selected with the highest PhaseHypoScore of 1.31. Corresponding to their inter-site distances, comparable pharmacophores are assembled through a tree-based partitioning technique [14]. This process helps to attain common pharmacophores. Table 2 and Table 3 display the inter-site angle and distances of the model.

**Figure 2:**
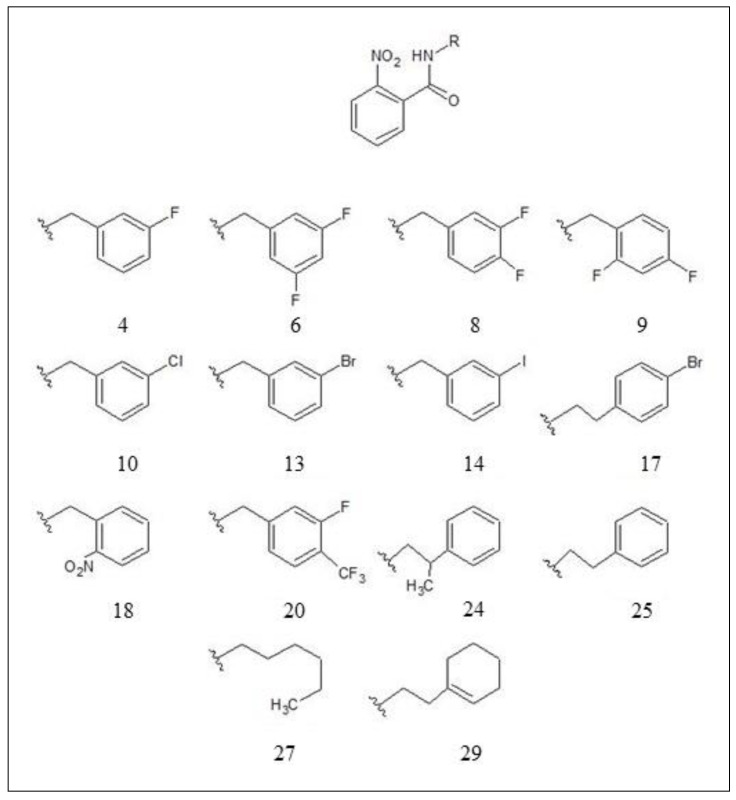
Inhibitor structures used for the generation of the selected hypothesis (ADHRR) [17].

The result of the survival inactive scores was used to pick out the best hypothesis model, which separates the active compounds from the inactive ones. Inhibitor structures used for the modelling of the selected hypothesis are shown in Figure 2. IC50 values of the nine actives, inhibitors 4, 6, 8, 10, 13, 14, 17, 18, and 25, for the selected hypothesis were 21 μM, 6.6 μM, 16 μM, 9 μM, 9.6 μM, 6.5 μM, 4.8 μM, 9.7 μM, and 21 μM, respectively. IC50 values of the five inactives, inhibitors 9, 20, 24, 27, and 29 were 45 μM, 35 μM, 37 μM, 40 μM, and 52 μM respectively [17].

**Figure 3:**
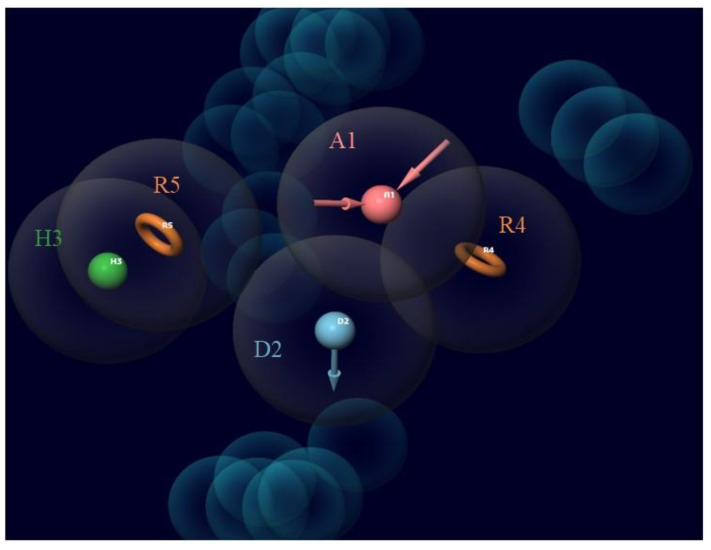
The five-feature pharmacophore model ADHRR as generated using PHASE illustrating the acceptor group (A1; pink), donor group (D2; blue), hydrophobic group (H3; green), and aromatic ring (R4, R5; orange). This model was created using known inhibitors of LasR. Almost all of the inhibitors used had these five features in common.

**Figure 4:**
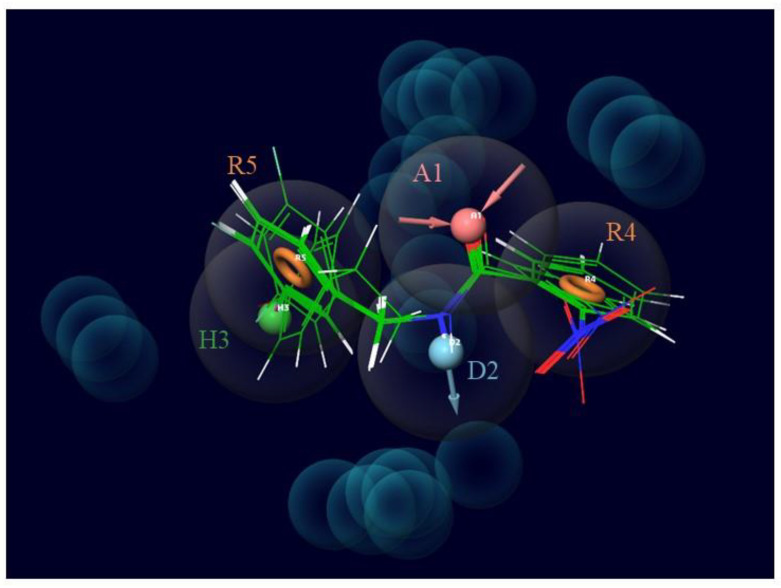
Superimposed images of active ligands (4, 6, 8, 10, 13, 14, 17, 18, and 25) with selected hypothesis ADHRR. The structural similarity in these ligands and their relationship with the hypothesis can be observed from this superimposed image.

One acceptor group (A), one donor group (D), one hydrophobic group (H), and two aromatic rings (ADHRR) are the five key features of the chosen CPH. Figure 3 displays the results of this hypothesis. Superimposed images of the active ligands with ADHRR are shown in Figure 4.

### 2.2. Phase Database Generation

From the 2373 FDA-approved compounds attained from the DrugBank, 1382 were obtained by applying Lipinski’s rule. Stereoisomers were generated by restricting specific chiralities and allowing variability for other chiral centers. At most, four low energy stereoisomers were retained and up to one low energy conformation for five to six membered rings were generated. Subsequently, the total number of compounds obtained was 2760, from which the average conformers generated per record was 49.08 and a total of 135,460 conformations were obtained.

### 2.3. Screening Using a Pharmacophore Model

**Figure 5:**
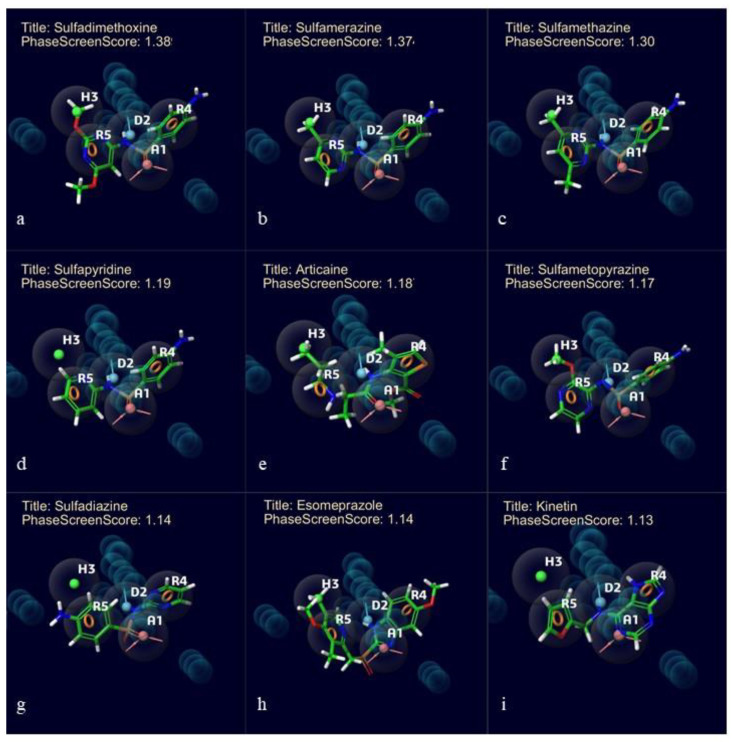
Superimposed images of screened hit molecules from the database with the selected hypothesis ADHRR. (**a**) Sulfadimethoxine, (**b**) sulfamerazine, (**c**) sulfamethazine, (**d**) sulfapyridine, (**e**) articaine, (**f**) sulfametopyrazine, (**g**) sulfadiazine, (**h**) esomeprazole, and (**i**) kinetin. PhaseScreenScore are mentioned along with the title of the compounds.

To find possible LasR-LBD inhibitors, the pharmacophore model (ADHRR 3) generated was employed to screen the phase database. For every ligand from the database in this study, the sites of the hypothesis were compared against a pre-computed set of conformers [14]. The molecules were compared to at least four sites of the hypothesis, hence four out of five sites were matched. From the 135,460 molecules examined, top ten compounds were prepared for the docking to analyze their binding ability. Phase screen score was used for finding out the top ten compounds. Values of the phase screen score are mentioned in Figure 5. These molecules have the essential features required to bind to LasR-LBD. These compounds were considered for further examinations as they may have more potential to inhibit LasR-LBD. Superimposed images of top screened molecules with the hypothesis are shown in Figure 5.

### 2.4. Molecular Docking Using GLIDE

**Figure 6:**
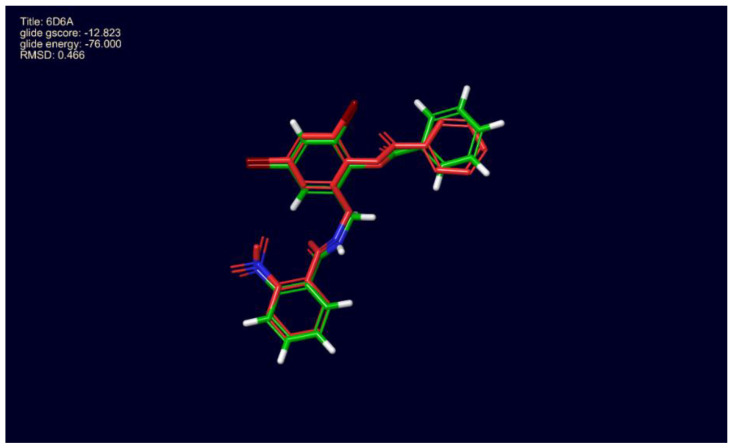
Docking protocol validation, 4-dibromo-6-{[(2-nitrobenzene-1-carbonyl)amino] methylphenyl benzoate redocked with LasR ligand-binding domain, 6D6A. Conformation of the docked molecule was found to be similar to the conformation of the inhibitor reported with 6D6A.

The docking protocol was validated by using a ligand from the LasR-LBD structure file, PDB ID: 6D6A, 2,4-dibromo-6-{[(2-nitrobenzene-1-carbonyl)amino]methyl}phenyl benzoate, for redocking (Figure 6). 6D6A is a high resolution (1.9 Å) X-ray structure of the complete LasR-LBD domain of *P. aeruginosa*. We found that the docked ligand occupied a similar position at the binding site of LasR-LBD. Root mean square deviation (RMSD) of the docked ligand was found to be 0.46 Å when compared with the ligand in the crystal structure.

**Figure 7:**
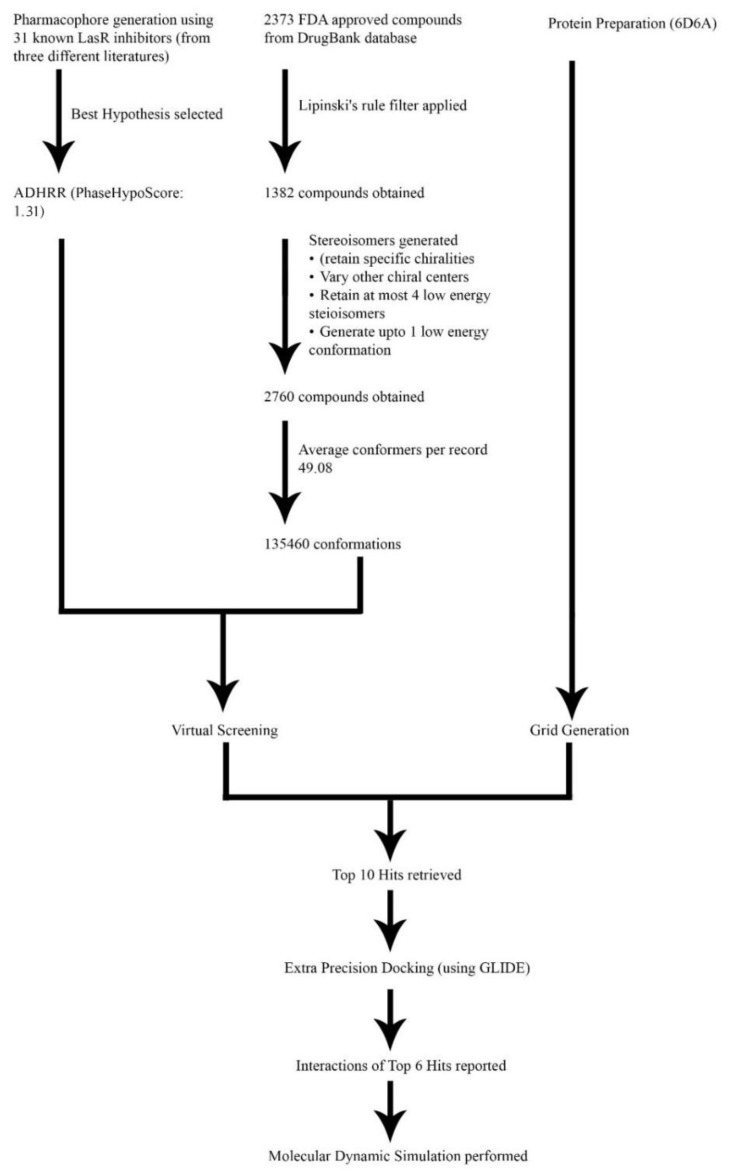
Graphical representation of Virtual Screening workflow illustrating the steps followed for hit identification.

**Figure 8:**
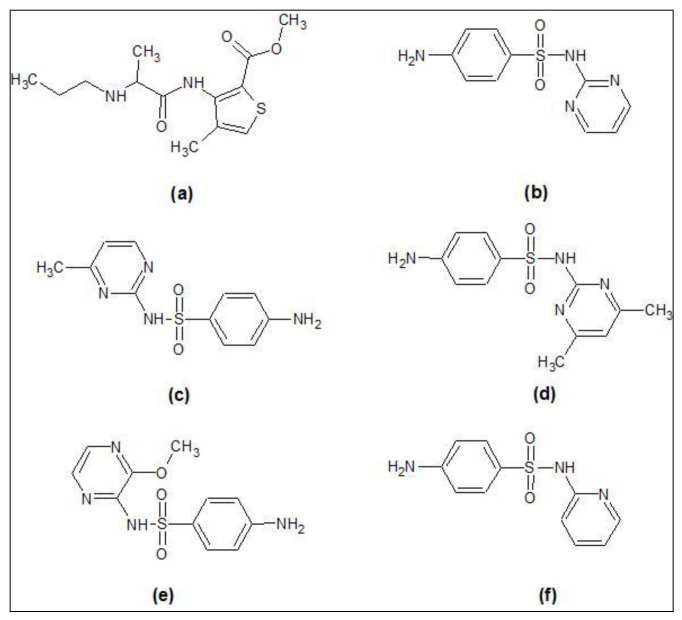
2D structures of the screened hit compounds (**a**) articaine, (**b**) sulfadiazine, (**c**) sulfamerazine, (**d**) sulfamethazine, (**e**) sulfametopyrazine, and (**f**) sulfapyridine.

At first, the nine reference ligands, actives of the selected CPH, were docked to the LasR-LBD following the calculation of their glide energies and scores. After that, the top ten hit molecules from the screening results were docked in the same binding site. Six hits with glide scores comparable to the known LasR-LBD inhibitors were attained from the results. These included articaine, sulfametopyrazine, sulfadiazine, sulfamethazine, sulfamerazine, and sulfapyridine. The glide score of sulfamerazine was −9.68 kcal/mol, which was greater than −9.28 kcal/mol, the glide score of the reference ligand 17. Figure 7 represents the complete process of virtual screening and docking. Figure 8 shows the 2D structures of hit molecules. Glide score and glide energy of the top seven reference ligands and the six best hits are shown in Table 4.

### 2.5. Interaction Studies of Screened Hit Molecules

**Figure 9:**
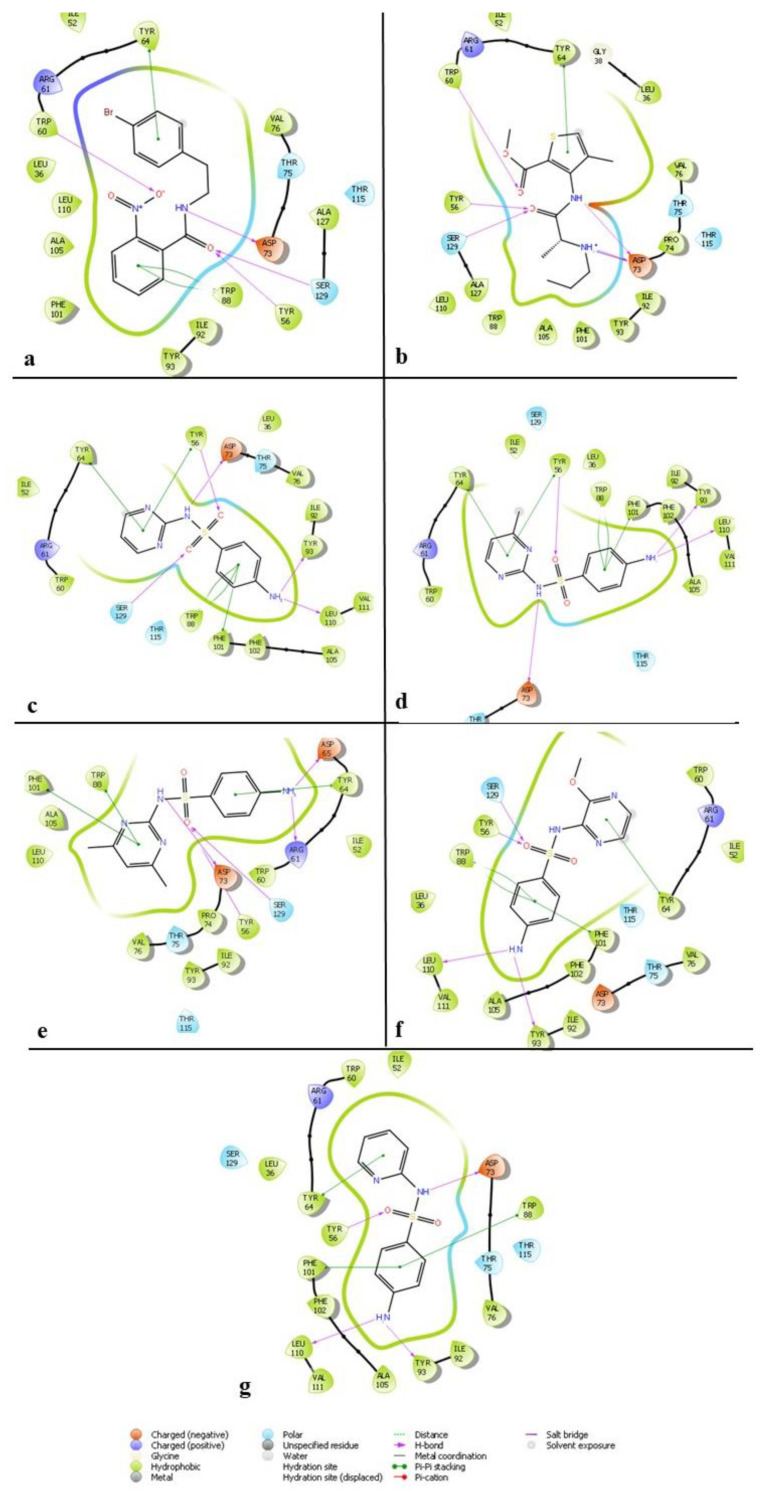
Ligand interaction diagram of screened hits to the active site of ALK with (**a**) 17, (**b**) articaine, (**c**) sulfadiazine, (**d**) sulfamerazine, (**e**) sulfamethazine, (**f**) sulfametopyrazine, and (**g**) sulfapyridine.

The ligand interaction diagram (LID) of the Schrödinger suite helped to examine the reference inhibitors and hit molecules’ interaction patterns (Table 5 and Table 6). Figure 9 shows the results obtained. The hydrogen bond interactions help to stabilize the ligand greatly and are represented by purple lines in the figure. Along with these H-bonds, pi-pi interactions are also substantial in the binding of the ligand. Hence, these two major interactions are critically observed in the hit molecules. Figure 9a shows that the LasR-LBD-17 complex, which was analyzed using LID, forms four H-bond interactions, and two pi-pi stacking interactions. The pi-pi stacking interactions were formed by Trp88 and Tyr64, whereas Trp60, Ser129, Tyr56, and Asp73 formed H-bonds. Reported literature emphasizes the significance of Asp73, Ser129, and Tyr56 in maintaining the stability of the ligand [18]. Additionally, Figure 9b–g displays the results of the analyses of the hit molecules, as well. Evidently, sulfamerazine formed four H-bond interactions while articaine formed five. The pi-pi stacking interactions that strengthen the protein-ligand binding when checked were present, with four such interactions for sulfamerazine and one for the articaine ligand [18].

### 2.6. Molecular Mechanics—General Born Surface Area (MM-GBSA) Estimation

The solvent effect on the interaction between LasR-LBD and top ranked docked compound was evaluated by determining the molecular mechanics—general born surface area (MM-GBSA). Table 4 shows MM-GBSA of compounds. The MM-GBSA of known inhibitors was recorded between −74.45 kcal/mol and −80.50 kcal/mol. Amongst the screened drugs, the MM-GBSA of sulfamerazine was found to be the lowest (−52.74 kcal/mol). Altogether, the MM-GBSA results suggest the formation of a stable complex between LasR-LBD and sulfamerazine. Further, MD simulation was used to estimate the stability of the LasR-LBD-sulfamerazine complex.

### 2.7. Molecular Dynamics Simulation

**Figure 10:**
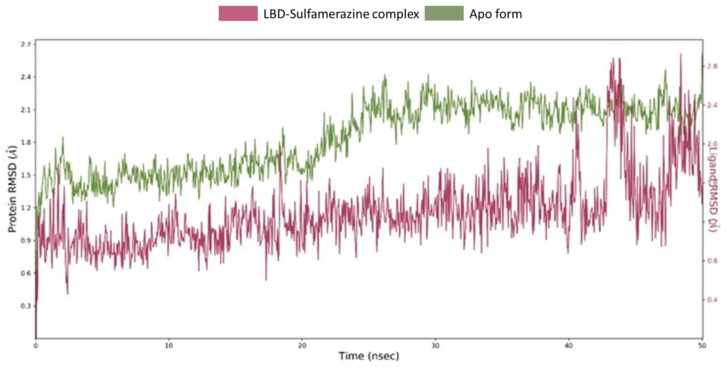
Root mean square deviations (RMSDs) trajectories derived from molecular dynamics (MD) simulation of the LBD-sulfamerazine complex apo form (no ligand). The system is well equilibrated as the average deviation was observed to follow the similar pattern in both the trajectories during the latter half of the MD simulation. The complex form showed higher RMSD during 40–45 ns, however, it converged for the last 5 ns, forming a stable complex with sulfamerazine.

MD simulation is used for the estimation of dynamics and stability of the ligand-protein complex. The complex of sulfamerazine-LasR-LBD obtained from the docking was subjected to MD simulation. Total duration of the simulation was 50 ns, Figure 10. It is evident that the system was well equilibrated as the root mean square deviations (RMSD) deviation followed the similar trajectory during the the latter half of the MD simulation, and the complex form trajectory tended to converge with that of the apo form during the last 5 ns of the simulation. In the first half of the simulation, the RMSD variation remained well below 0.3 Å, ranging between 0.9–1.2 Å for the complex and 1.2–1.5 Å for the apo form (without ligand). In the latter half, deviation again remained below 0.3 Å followed by a surge of 1 Å. The RMSD of the protein apo form remained within the upper limit of 2.0 Å. Also, the RMSD of sulfamerazine showed variation within 0.3–2.4 Å. In the last 5 ns, the LBD of both apo and the complex with sulfamerazine were observed to converge. Collectively, these results propose the formation of a stable complex between LasR-LBD and sulfamerazine upon the formation of favorable interactions with key amino acid residues.

**Figure 11:**
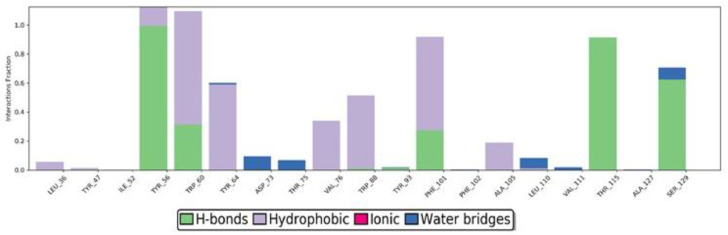
Ligand-protein interactions duration during simulation. Interaction fraction of more than 0.8 observed with amino acids TYR_56, TRP_60, PHE_101, and THR_115.

**Figure 12:**
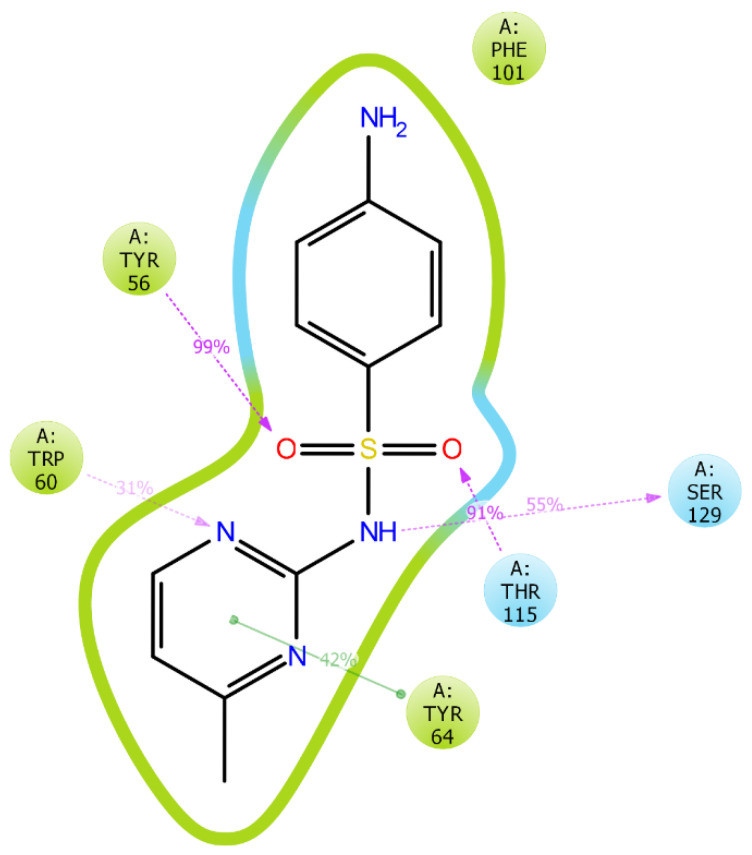
Atomic contributions in ligand-protein interactions. Hydrogen bonding observed with TYR56, TRP60, THR115, and SER129. Hydrophobic interactions observed with TYR64. Purple: Hydrogen bond, Green: Hydrophobic interaction.

Protein interactions with the ligand were monitored throughout the simulation. These interactions were categorized by their types and summarized, as shown in Figure 11 and Figure 12. Protein-ligand interactions (or “contacts”) were categorized into four types: hydrogen bonds, hydrophobic, ionic, and water bridges. The stacked bar charts are normalized throughout the trajectory, for example, a value of 0.7 suggests that for 70% of the simulation time, the specific interaction is maintained. Values over 1.0 are possible as some protein residues may make multiple contacts of the same subtype with the ligand. The atomic contributions of the ligand in these interactions can be seen in Figure 11. Interaction analysis of LasR-LBD and the sulfamerazine complex after MD simulation showed that it binds to the allosteric binding site of the enzyme. The complex was stabilized by two hydrogen bonds with Tyr56, Trp60, Thr115, and Ser129 along with one hydrophobic interaction with Tyr64 (Figure 11 and Figure 12).

Hydrogen bonds play a significant role in ligand binding. Consideration of hydrogen-bonding properties in drug design is important because of their strong influence on drug specificity, metabolization, and adsorption. Hydrogen bonds between a protein and a ligand can be further broken down into four subtypes: backbone acceptor; backbone donor; side-chain acceptor; and side-chain donor.

## 3. Discussion

*P. aeruginosa*, a Gram-negative bacterium, is responsible for urinary tract infections, nosocomial pneumonia, and bloodstream infections [19]. The ability of *P. aeruginosa* to produce biofilms [20,21] and increased antibiotic resistance has become the driving force to find new therapies that can address this issue. Recent efforts have been focused in developing antipathogenic strategies by decreasing bacterial virulence through QS systems [22,23]. Evidence suggested the attenuation of pathogenicity of *P. aeruginosa* through inhibition of the LasR QS system [22,24,25,26]. Therefore, impeding QS in *P. aeruginosa* by the use of LasR inhibitors is a promising strategy for the treatment of infections [22]. Different groups have identified a series of LasR inhibitors using traditional methods from natural resources [22,27,28,29]. Novel computer-aided drug designing can address the limitations of traditional methods [18,30]. This brings a new opportunity for the designing of LasR inhibitors, which can reduce pathogenicity, virulence, and resistance rather than directly inhibiting the bacterial growth.

The core objective of this study was to find out potential LasR-LBD inhibitors from already approved drugs through pharmacophore-based virtual screening. A total of 1382 drug molecules and 135,460 conformations were screened, out of which the top ten compounds were docked against LasR-LBD. Molecular docking results showed six compounds, namely, articaine, sulfametopyrazine, sulfadiazine, sulfamethazine, sulfamerazine, and sulfapyridine, with docking scores comparable to the known LasR-LBD inhibitors that were used for the development of the pharmacophore hypothesis. The docking score of sulfamerazine was −9.68 kcal/mol, which was greater than −9.28 kcal/mol, the docking score of one of the reference ligands. The drug molecule with the highest binding affinity, sulfamerazine, was further utilized for molecular dynamics simulation to check the stability of binding interactions. Collectively, these results proposed the formation of a stable complex between LasR-LBD and sulfamerazine upon the formation of favorable interactions with key amino acid residues.

The analysis of the ligand binding interaction revealed the involvement of active site residues, i.e., Try56, Trp60, Tyr64, Asp73, Trp88, Tyr93, Phe101, Leu110, and Ser129. This result was found to be consistent with the previously conducted research on LasR inhibitors [13,31,32]. Several studies have been conducted for finding potential inhibitors of LasR. They focused mostly on traditional remedies [31], 147 approved drugs and natural compounds from SuperNatural and SuperDrug databases [32], ZINC database [33,34], TimTec’s Natural Derivatives Library [35], and traditional Chinese medicines [36]. In contrast, our research study focused on FDA-approved drugs from drugbank database [37]. Since these compounds are already approved for human use, there is a better chance of developing antipathogenic therapy in a shorter duration.

In comparison to our study, only one of the previous studies used a pharmacophore modelling technique for finding potential LasR inhibitors [33]. In this study, the native ligand of LasR was used for searching the two most structurally similar compounds from the PubChem database. These three compounds were then used for developing the pharmacophore model. In contrast to this, we used thirty-one previously reported LasR inhibitors with varying IC50 values. Three different hypotheses were developed out of which one was selected based on the survival inactive scores, which separates the active compounds from the inactive ones.

Our docking and simulation results showed that these compounds can further be tested in vitro. Also, these compounds can serve as lead compounds for designing or optimizing LasR inhibitors.

## 4. Materials and Methods

### 4.1. Compound Data Set

For this study, the x-ray crystal structure of LasR-LBD (PDB code: 6D6A) was downloaded from the Protein Data Bank having a resolution of 1.9 Å [38]. Three different pharmacophore hypotheses (PH) were modelled from the previously reported thirty-one LasR-LBD inhibitors of varying IC50 values [15,16,17]. To perform virtual screening, 2373 FDA-approved compounds from DrugBank (release 5.1.4 uploaded on 02-07-2019) were downloaded in structure-data file (SDF) file format [37].

### 4.2. Protein Preparation

The protein preparation wizard of Maestro, schrödinger graphical user interface, was used for protein preparation of LasR-LBD [39]. The original protein structures were altered by assigning bond orders, incorporating H atoms, forming disulphide bonds, and fixing the residue charges [40]. Water molecules beyond 5 Å of the allosteric binding site were removed. These structures were minimized with the help of the OPLS 2005 force field [39,41]. This altered protein structure was ultimately used for docking studies [39]. The binding pocket was identified by the coordinates of the already bound ligand (TP-1).

### 4.3. Phase Database Creation

The drugbank database of FDA-approved drugs contained 2373 compounds in the ready-to-dock SDF format. These compounds were shortlisted into 1382 compounds by applying Lipinski’s rule of five as a prefilter and prepared in LIGPREP [42]. The ionization states of the compounds were generated at pH 7.0 ± 2.0 with the help of EPIK [43] followed by the removal of salts from ligands. A maximum of thirty-two stereoisomers were generated for a compound. An OPLS3 (optimized potentials for liquid simulations) force field was used to minimize the energy of each compound with default parameters. Ligand conformers were generated using the conformers generation option in the create phase database wizard.

### 4.4. Pharmacophore Generation

The PHASE(PH) module of the Schrödinger package was used for the preparation of the pharmacophore [44]. The PH essentially provides details about the minimum and necessary structural features that are required to bind to the target protein. The generation of PH in this study relied upon the LasR-LBD inhibitors present. Before the generation of the pharmacophore, thirty-one LasR-LBD inhibitors were processed using LIGPREP [42] with EPIK for expanding the protonation and tautomeric states at physiological pH 7.0 ± 2.0 [41]. This was followed by the application of the OPLS3 force field. Conformers were also generated for ligands [45]. All the ligands were aligned, and a multiple ligand-protein approach was used in which both ligands and proteins were analyzed for the formation of a common pharmacophore hypothesis (CPH). The set of six built-in pharmacophore features provided by PHASE are: aromatic ring (R), positively ionizable (P), negatively ionizable (N), hydrogen bond donor (D), hydrogen bond acceptor (A), and hydrophobic group (H). The survival active, as well as survival inactive scores, were evaluated once the pharmacophore hypotheses were generated based on a set of SMART patterns for every feature [44].

### 4.5. Phase Screening

Virtual screening was performed using a previously created phase database against the generated pharmacophore model. This screening results in the identification of compounds having structural similarities with the reported ligands based on the CPH. Top results of phase screening were further used in the molecular docking study.

### 4.6. Virtual Screening Using the Molecular Docking Study

Using LasR-LBD (PDB code: 6D6A) with a scaling factor with 1.0 Å, a receptor grid was made having coordinates 57.49, 16.00, and 6.65 for x, y, and z, respectively. The size of the outer box was set to 26.11 for all three dimensions, x, y, and z. GLIDE from the Schrödinger interface was used for these docking studies [46]. The extra precision (XP) method was used for molecular docking study.

#### Docking Validation

The validity of the docking protocol was confirmed by performing XP docking of the X-ray crystal structure ligand TP-1 homolog at the allosteric binding site of LasR-LBD and comparing the RMSDs between the docked pose and the crystal structure pose of the ligand.

### 4.7. Molecular Mechanics—Generalized Born Surface Area (MM-GBSA) Calculations

The solvent effect on the binding free energies of the compounds selected for XP docking was estimated. Implicit solvation and molecular mechanics force fields were used through the MM-GBSA method of PRIME [47]. Binding energy calculations were generated using pose viewer file. PRIME local optimization feature was used to minimize the docked poses. The MM-GBSA continuum solvent model was used to compute binding free energies of the docked compounds. This solvent model incorporates the VSGB solvent model [48], the OPLS3 force field [49], and rotamer search algorithms [50].

### 4.8. Molecular Dynamics Simulation (MD)

MD simulation was used to determine the stability of the docked complex as described previously [51]. DESMOND was used for the 50 ns MD simulation of the protein-ligand complex with minimum MM-GBSA binding energy [52]. The orthorhombic simulation box with a TIP3P explicit water model was prepared using a system builder panel. The 10 Å distance was maintained between the boundary of the simulation box and the protein surface. The system was neutralized, and 150 mM NaCl was added to maintain the isosmotic salt environment. The system was minimized with 2000 iterations. A 50 ns MD simulation was performed on the minimized system using the NPT (normal pressure and temperature) ensemble at 300 °K and 1.01 bars with the default setting of relaxation before simulation. The Martyna–Tobias–Klein barostat [53] and Nose–Hoover Chain thermostat [54] were used to maintain the pressure and temperature, respectively. The energy and structure were recorded and saved in the trajectory file at every 10 ps, and a time step of 2 fs was considered during the simulation. MAESTRO was used for the inspection of trajectories and three-dimensional structures [55].

## 5. Conclusions

This research adopted a combined structure and ligand-based VS approach to detect potential LasR inhibitors from the FDA-approved drugs for their repurposing. Fourteen LasR-LBD inhibitors generated a five-point CPH (ADHRR). An FDA-approved subset of the DrugBank database was used to create a phase database that was then screened based on ADHRR. Docking was performed for the hit molecules, and it was revealed that six of these hits had glide scores comparable to their reference ligands. MM-GBSA of sulfamerazine indicated a stable complex of the drug with LasR-LBD. The MD simulations carried out for the sulfamerazine-LasR-LBD complex almost reproduced the docking interaction pattern during trajectory analysis. Hydrogen bonding was observed for more than 70% of the simulation duration. Finally, it was concluded that sulfamerazine showed the highest binding affinity to LasR-LBD and exhibited strong and stable binding interactions during MD simulation.

## Figures and Tables

**Table 1:** Pharmacophore hypothesis identified using the PHASE module.

Inhibitors Used	Pharmacophore Hypothesis	PhaseHypoScore	Reference of Inhibitors
6	AAADHHR	1.11	[15]
11	AADHHRR	1.05	[16]
14	ADHRR	1.31	[17]

**Table 2:** Inter-pharmacophoric site measurements of model ADHRR.

Site 1	Site 2	Distance (Å)	Site 1	Site 2	Distance (Å)
A1	D2	3.18	D2	R4	3.53
A1	H3	6.49	D2	R5	3.99
A1	R4	3.74	H3	R4	7.35
A1	R5	4.46	H3	R5	2.74
D2	H3	5.01	R4	R5	6.61

**Table 3:** Inter-pharmacophoric angle measurements of model ADHRR.

Site 1	Reference	Site 2	Angles (°)	Site 1	Reference	Site 2	Angles (°)
A1	D2	H3	16.1	D2	A1	H3	48.8
A1	D2	R4	48.6	D2	A1	R4	51.8
A1	D2	R5	70.4	D2	A1	R5	50.4
A1	H3	R4	21.4	H3	A1	R4	81.2
A1	H3	R5	23.8	H3	A1	R5	24.9
A1	R4	R5	50.6	R4	A1	R5	97.0
D2	H3	R4	20.1	H3	D2	R4	117.3
D2	H3	R5	34.1	H3	D2	R5	45.7
D2	R4	R5	36.1	R4	D2	R5	112.2
H3	R4	R5	31.8	R4	H3	R5	40.6

**Table 4:** Docking scores and energy involvement of reference LasR inhibitors and screened hit molecules against LasR-LBD.

Ligands	Glide Gscore (kcal/mol)	Glide Energy (kcal/mol)	MMGBSA dg Bind (kcal/mol)
**Results of reference LasR inhibitors**			
8	−10.24	−53.28	−75.43
10	−10.17	−55.49	−80.50
6	−10.10	−52.53	−75.53
13	−10.06	−56.90	−79.33
14	−10.01	−56.38	−79.76
4	−9.95	−52.45	−74.45
17	−9.28	−56.20	−76.42
**Results of LasR inhibitors**			
Articaine	−9.01	−41.63	−46.27
Sulfamerazine	−9.68	−47.48	−52.74
Sulfametopyrazine	−8.37	−48.73	−52.71
Sulfapyridine	−8.07	−45.25	−51.35
Sulfadiazine	−8.27	−45.08	−44.23
Sulfamethazine	−7.86	−50.23	−51.16

**Table 5:** Interaction analysis of reference LasR inhibitors against LasR-LBD.

S. No.	Compound	Number of H Bonds	Interacting Atoms of Protein-Ligand Complex	Distance (Å)
1	8	4	TYR 56…Lig(O)	1.81
			Lig(NH)…ASP 73	1.82
			TRP 60…Lig(O)	2.11
			SER 129…Lig(O)	2.13
2	10	4	TRP 60…Lig(O)	2.07
			TYR 56…Lig(O)	1.97
			SER 129… Lig(O)	2.08
			Lig(NH)…ASP 73	1.73
3	6	4	TRP 60…Lig(O)	2.21
			TYR 56…Lig(O)	1.88
			SER 129…Lig(O)	2.15
			Lig(NH)…ASP 73	1.79
4	13	4	TYR 56…Lig(O)	1.86
			SER 129…Lig(O)	2.12
			Lig(NH)…ASP 73	1.83
			TRP 60…Lig(O)	2.10
5	14	4	TYR 56…Lig(O)	1.97
			SER 129…Lig(O)	2.08
			Lig(NH)…ASP 73	1.74
			TRP 60…Lig(O)	2.08
6	4	4	SER 129…Lig(O)	2.13
			TYR 56…Lig(O)	1.85
			Lig(NH)…ASP 73	1.82
			TRP 60…Lig(O)	2.14
7	17	4	TRP 60…Lig(O)	2.18
			Lig(NH)…ASP 73	1.86
			SER 129…Lig(O)	2.15
			TYR 56…Lig(O)	1.89

**Table 6:** Interaction analysis of screened hit molecules against LasR-LBD.

S. No.	Compound	Number of H Bonds	Interacting Atoms of Protein-Ligand Complex	Distance (Å)
1	Articaine	5	TRP 60…Lig(O)	2.64
			Lig(NH)…ASP 73	1.73
			TYR 56…Lig(O)	2.24
			SER 129…Lig(O)	2.06
			Lig(NH_2_^+^)…ASP 73	2.01
2	Sulfamerazine	4	Lig(NH)…ASP 73	4.67
			TYR 56…Lig(O)	5.68
			Lig(NH_2_)…TYR 93	5.71
			Lig(NH_2_)…LEU 110	5.18
3	Sulfametopyrazine	4	SER 129…Lig(O)	2.00
			TYR 56…Lig(O)	1.82
			Lig(NH_2_)…LEU 110	2.16
			Lig(NH_2_)…TYR 93	2.26
4	Sulfapyridine	4	Lig(NH)…ASP 73	2.46
			TYR 56…Lig(O)	2.06
			Lig(NH_2_)…LEU 110	2.52
			Lig(NH_2_)…TYR 93	2.19
5	Sulfadiazine	5	Lig(NH)…ASP 73	2.25
			TYR 56…Lig(O)	1.70
			SER 129…Lig(O)	1.95
			Lig(NH_2_)…TYR 93	1.88
			Lig(NH_2_)…LEU 110	2.43
6	Sulfamethazine	5	Lig(NH)…ASP 73	1.80
			TYR 56…Lig(O)	2.58
			SER 129…Lig(O)	2.46
			Lig(NH_2_)…ASP 65	2.74
			Lig(NH_2_)…ARG 61	2.79
